# Dopamine Modulates Adaptive Prediction Error Coding in the Human Midbrain and Striatum

**DOI:** 10.1523/JNEUROSCI.1979-16.2016

**Published:** 2017-02-15

**Authors:** Kelly M.J. Diederen, Hisham Ziauddeen, Martin D. Vestergaard, Tom Spencer, Wolfram Schultz, Paul C. Fletcher

**Affiliations:** ^1^Department of Psychiatry and; ^2^Department of Physiology, Development, and Neuroscience, University of Cambridge, Cambridge CB2 3DY, United Kingdom,; ^3^Wellcome Trust MRC Institute of Metabolic Science, Cambridge Biomedical Campus, Cambridge, CB2 0QQ, United Kingdom, and; ^4^Cambridgeshire and Peterborough Foundation Trust, Fulbourn Hospital, Cambridge, CB21 5EF, United Kingdom

**Keywords:** adaptation, dopamine, fMRI, pharmacological intervention, prediction errors, reward

## Abstract

Learning to optimally predict rewards requires agents to account for fluctuations in reward value. Recent work suggests that individuals can efficiently learn about variable rewards through adaptation of the learning rate, and coding of prediction errors relative to reward variability. Such adaptive coding has been linked to midbrain dopamine neurons in nonhuman primates, and evidence in support for a similar role of the dopaminergic system in humans is emerging from fMRI data. Here, we sought to investigate the effect of dopaminergic perturbations on adaptive prediction error coding in humans, using a between-subject, placebo-controlled pharmacological fMRI study with a dopaminergic agonist (bromocriptine) and antagonist (sulpiride). Participants performed a previously validated task in which they predicted the magnitude of upcoming rewards drawn from distributions with varying SDs. After each prediction, participants received a reward, yielding trial-by-trial prediction errors. Under placebo, we replicated previous observations of adaptive coding in the midbrain and ventral striatum. Treatment with sulpiride attenuated adaptive coding in both midbrain and ventral striatum, and was associated with a decrease in performance, whereas bromocriptine did not have a significant impact. Although we observed no differential effect of SD on performance between the groups, computational modeling suggested decreased behavioral adaptation in the sulpiride group. These results suggest that normal dopaminergic function is critical for adaptive prediction error coding, a key property of the brain thought to facilitate efficient learning in variable environments. Crucially, these results also offer potential insights for understanding the impact of disrupted dopamine function in mental illness.

**SIGNIFICANCE STATEMENT** To choose optimally, we have to learn what to expect. Humans dampen learning when there is a great deal of variability in reward outcome, and two brain regions that are modulated by the brain chemical dopamine are sensitive to reward variability. Here, we aimed to directly relate dopamine to learning about variable rewards, and the neural encoding of associated teaching signals. We perturbed dopamine in healthy individuals using dopaminergic medication and asked them to predict variable rewards while we made brain scans. Dopamine perturbations impaired learning and the neural encoding of reward variability, thus establishing a direct link between dopamine and adaptation to reward variability. These results aid our understanding of clinical conditions associated with dopaminergic dysfunction, such as psychosis.

## Introduction

Optimal decision-makers choose options associated with the best outcomes. A powerful strategy for learning the value of different options is to update values in response to prediction errors (PEs), that is, the mismatch between predicted and actual outcomes (Sutton and Barto, 1998). Although larger PEs might suggest a greater need to update values, the size of the PE is meaningless without an estimate of its precision (i.e., its inverse variance) (Preuschoff and Bossaerts, 2007). Repeatedly modifying predictions in an attempt to minimize markedly fluctuating PEs would be suboptimal (Nassar et al., 2010). To avoid unstable learning, it is thus essential to compare PEs to the expected fluctuation in reward value (Preuschoff and Bossaerts, 2007), and update values more when PEs with higher precision are encountered. The brain is thought to implement this by adaptively coding PEs relative to reward variability.

PEs are coded by midbrain dopamine neurons (Schultz et al., 1997), and adaptive PE coding has been demonstrated in monkey midbrain dopamine neurons (Tobler et al., 2005). Such neural adaptation sensitizes the detection of smaller PEs when the outcome variability (i.e., its SD) is smaller (Kobayashi et al., 2010). We have recently shown that humans weight PEs relative to reward variability to guide learning. This is reflected in higher learning rates when reward variability is lower (Diederen and Schultz, 2015). The activity in the midbrain and ventral striatum, areas that are part of the mesolimbic dopaminergic pathway, is sensitive to this reward PE adaptation and the degree of neural adaptation correlates with behavioral adaptation, thus establishing a direct relationship between neural and behavioral measures of adaptation (Diederen et al., 2016).

Although the above observations strongly suggest a critical role for dopamine in adaptive coding in humans similar to monkeys, thus far there is no direct evidence to support this. We therefore sought to more directly investigate the role of dopamine in adaptive PE coding in humans, using fMRI in conjunction with a dopamine D2 antagonist (sulpiride) and D2 agonist (bromocriptine), to produce perturbations of dopaminergic function in healthy volunteers engaged in a task requiring PE adaptation. We administered dopaminergic agents with high affinity for D2 receptors as these receptors are densely distributed in the mesolimbic dopaminergic pathway, which is implicated in PE coding (Grace, 2002; Pizzagalli et al., 2008). D2 receptor density is highest in the basal ganglia, but these receptors are also expressed in the midbrain (Aghajanian and Bunney, 1977; Lacey et al., 1987; Mercuri et al., 1992). We used a previously validated task (Diederen and Schultz, 2015; Diederen et al., 2016) that required participants to predict the magnitude of rewards drawn from distributions with different SDs. On each trial, an explicit prediction and outcome are available, from which trial-by-trial PEs can be obtained. In addition to examining learning performance, we can obtain a measure of behavioral adaptation by fitting a computational model to the observed predictions, and neural adaptation, which is reflected in decreased PE coding slopes as SD increases. The study design therefore permitted the examination of dopamine agonism and antagonism on learning performance and behavioral and neural adaptation of PEs.

We found that the dopamine antagonist sulpiride reduced adaptive PE coding in the midbrain and ventral striatum, suggesting that normal dopaminergic function is critical for this process. Whereas this effect was apparent across positive and negative PEs in the midbrain, sulpiride selectively impaired adaptive coding of positive PEs in the ventral striatum. Sulpiride also impaired learning performance in parallel with adaptive coding, supporting the hypothesis that PE adaptation benefits performance.

## Materials and Methods

### 

#### 

##### Participants.

Sixty-three healthy individuals were recruited into this pharmacological fMRI study through local advertisements. Participants consisted of university students and academics (*N* = 38) as well individuals from the local community (*N* = 21). The majority of individuals from the local community (17 of 21) had obtained an undergraduate university degree or higher. All participants were fluent English speakers, had no history of neurological or psychiatric illness or drug abuse, and were not using any psychoactive medication. The study was approved by the Local Research Ethics Committee of the Cambridgeshire Health Authority. Written informed consent was obtained from all participants.

##### Pharmacological perturbation.

In a double-blind placebo-controlled design, participants received a single oral dose of bromocriptine 2.5 mg (dopamine D2 agonist; *N* = 20), sulpiride 600 mg (D2 antagonist; *N* = 22), or placebo (*N* = 21). We used a between-subjects design as learning during initial sessions can interact with learning during later sessions in a within-subjects design. Because adaptive coding effects tend to be subtle, we used higher doses of bromocriptine and sulpiride compared with previous studies (Cools et al., 2009; Dodds et al., 2009; Morcom et al., 2010; Medic et al., 2014). Although a higher incidence of side effects might be expected with high doses of sulpiride, doses of 800 mg have been used in healthy controls without significant side effects (Takano et al., 2006; Eisenegger et al., 2014).

##### Study procedure.

Participants attended the Clinical Research Facility at Cambridge Biomedical Campus for a single study session. They arrived at the Clinical Research Facility between 0800 and 0900, except for one participant who arrived at 1100. Participants were informed that they would receive breakfast at the Clinical Research Facility and to abstain from food on the morning of the study, unless fasting would make them feel unwell. Upon arrival and provision of consent, participants gave a urine sample to test for recent drug use, and for pregnancy in the female participants. Weight, height, blood pressure, body temperature, and pulse rate were measured. Participants completed visual analog scales to indicate their mood and alertness at the start of the study (Bond and Lader, 1974), and a trained psychiatrist obtained a baseline measurement for the rating of extrapyramidal side effects (Simpson and Angus, 1970). The participants then completed the National Adult Reading Test (Nelson and Willison, 1991) and digit span backwards (Wechsler, 1958) to measure verbal IQ and working memory.

Thirty minutes after arrival, participants received either an experimental drug or placebo, along with 10 mg of the peripheral dopamine antagonist domperidone to prevent nausea, in line with reported procedures (Morcom et al., 2010; Medic et al., 2014). This was critical to the double blinding as nausea would be indicative of the administration of an active drug.

After drug administration, participants received a standardized breakfast to minimize variability of drug absorption. Following this, participants filled out a number of personality questionnaires (not reported here) and completed training on the experimental task. The visual analog scales for mood and alertness, examination for extrapyramidal side effects, measurement of blood pressure, temperature and pulse rate, and blood sampling were repeated 2 h after dosing. We collected blood samples to allow for quantification of drug plasma levels to be able to check the effectiveness of our drug manipulations.

fMRI scans were acquired ∼2.5 h after dosing to capture the window of maximal drug effect. Bromocriptine reaches peak plasma levels 1–3 h after dose, with a half-life of ∼15 h, whereas sulpiride reaches its maximal plasma concentration ∼3 h after dose and has a plasma half-life of ∼12 h (Wiesel et al., 1980; Caley and Weber, 1995; Kvernmo et al., 2006; Medic et al., 2014). Participants received a flat fee of £50 for their participation plus up to £15 in prize money, depending in part on their performance on the task (see below).

##### Experimental task design.

**Figure 1.**
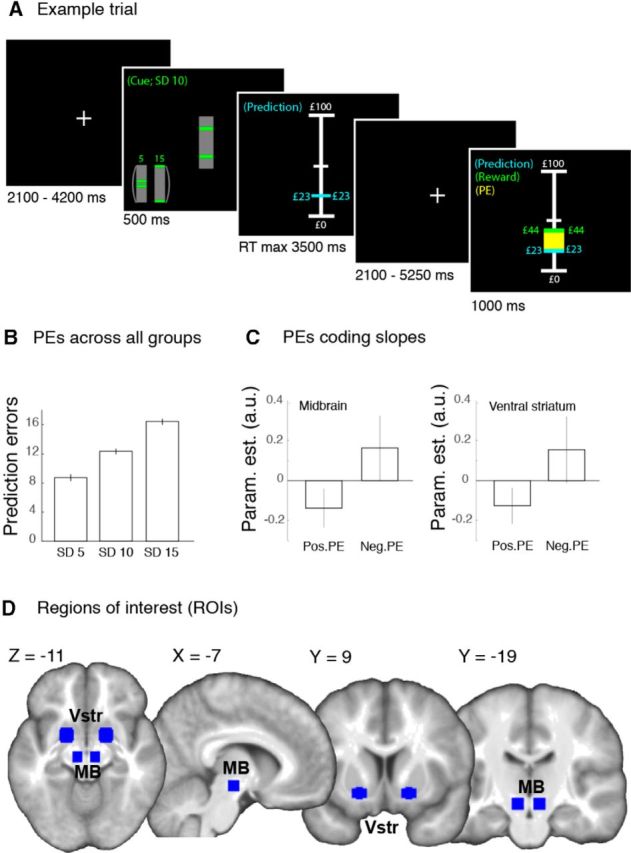
***A***, Participants predicted the magnitude of upcoming rewards as closely as possible from the past reward history. Vertical bar cues signaled whether rewards would be drawn from a distribution with small, medium, or large variability. After stating their prediction, participants received a reward, displayed in green. Yellow bar, spanning the distance between the predicted and the received reward, represents the reward PE. ***B***, The average (±SEM) PEs increased as SD increased, thus indicating that the experimental manipulation was successful. ***C***, The average (±SEM) magnitude of PE coding slopes increased for negative compared with positive PEs in the midbrain (left) and ventral striatum (right). ***D***, Midbrain and ventral striatal ROIs. To construct these ROIs, we drew spheres centered at MNI coordinates in the SN/VTA (−8, −18, −10 and 8, −18, −10) and ventral striatum (18, 1, −10 and 18, 1, −10) and their contralateral homologs that corresponded to areas displaying significant adaptive coding in an independent set of data (Diederen et al., 2016). The radii (6/8 mm for the midbrain/ventral striatum) were chosen to ensure that the spheres fell within the anatomical boundaries of the midbrain SN/VTA complex and the ventral striatum. a.u., arbitrary units; MB, Midbrain; Neg., Negative; Param. est., Parameter estimates; PE, prediction error; Pos., Positive; RT, reaction time; SD, standard deviation; Vstr, Ventral striatum.

During fMRI data acquisition, participants guessed the magnitude of upcoming rewards drawn from one of six pseudo-Gaussian distributions with a SD of £5, £10, or £15 and an expected value (EV) of £35 or £65 (31 trials per reward distribution) (Diederen and Schultz, 2015; Diederen et al., 2016). After each prediction, participants received a reward, yielding trial-by-trial PEs (Fig. 1*A*). Participants completed three task sessions, and every session used two reward distributions drawn pseudo-randomly from the six distributions. Importantly, we ensured that the two distributions in a session never had the same EV and/or SD.

Distributions were presented in short blocks of 4–6 trials. Explicit cues (i.e., Fig. 1*A*, gray vertical rectangles intersected by 2 horizontal green bars) signaled whether rewards would be drawn from a distribution with a small, medium, or large SD. Importantly, the cues only indicated the relative degree of variability and not the actual SD of the reward distributions, and they contained no information about the EV of the rewards. After cue presentation, the participants had 3500 ms to indicate their prediction of reward with a trackball mouse. The blue mouse cursor could be moved across a vertical scale that indicated the range of possible predictions (£0-£100). The starting position of the cursor varied randomly across trials so as to decorrelate prediction magnitude from scrolling distance. The vertical scale disappeared once the participants had indicated their prediction with a mouse click. After a variable delay of 2100–5250 ms (sampled from a uniform distribution), which was included to allow BOLD responses for prediction and reward to be differentiated, the received reward was displayed as a green line on the vertical scale, along with the participant's predicted reward on that trial. Furthermore, the PE was represented as a yellow bar spanning the distance between the predicted and received rewards. Failure to make a timely prediction led to omission of the reward. Inspection revealed that PEs increased as SD increased, indicating that the experimental manipulation was successful (*F*_(2,174)_ = 83.39, *p* < 0.001; Fig. 1*B*).

##### Task instructions.

We used a standardized tutorial, presented using MATLAB (The MathWorks), to instruct participants that the goal of the experiment was to predict the next reward as closely as possible from the past reward history. The tutorial informed the participants that rewards were drawn from “pots” (i.e., distributions) with small, medium, or large variability as indicated by the cues and that each task session would require them to alternatingly predict from one of two “pots.” Finally, we indicated that all changes in condition would be signaled using the bar cues so that participants were only unaware of the exact parameter values of the task (i.e., the frequency of alternation between the two distributions within a session as well as the SDs and EVs used).

##### Practice sessions.

Before the start of the fMRI scans, all participants completed a short motor task to familiarize themselves with the trackball mouse (Diederen and Schultz, 2015; Diederen et al., 2016). In addition, the participants completed two training sessions of the experimental task to ensure that they understood the task. The training sessions used reward distributions with an SD and EV that differed from those used in the fMRI task (i.e., SD, £7/£14 and EV, £30/£60).

##### Incentive compatibility.

To ensure that participants would indicate their true prediction of reward, 20% of the trials were control trials, which were pseudorandomly interspersed across the session. In these control trials, the pay-off depended on participants' performance (i.e., how close they were to the EV of the distribution; |prediction − EV|). Predictions within 1 and 2 SDs of the EV resulted in a pay-off of £7.50 and £5.00, respectively, whereas all other predictions led to a pay-off of £2.50. In the test trials (80%), the pay-off was a fraction (10%) of the reward drawn by the computer. While participants were informed beforehand of the presence of control trials in the task, critically the type of trial was only revealed at the outcome phase, when on the control trials the reward was indicated in red instead of green, thus encouraging participants to optimize their performance on all trials. Participants were told that, at the end of the experiment, one control and one test trial would be selected randomly and they would receive the money gained on these 2 trials as an additional payment. This design motivated the participants to consider rewards drawn by the computer as actual rewards. All analyses included the main test (80%) and the control trials (20%) as previous work has shown that participants use the reward history from all available trials to predict upcoming rewards and favor higher outcome trials (Diederen et al., 2016).

##### fMRI acquisition and preprocessing.

fMRI data were obtained at the Wolfson Brain Imaging Center, Cambridge, using a Siemens Trio 3T MRI scanner. We acquired 360 multiecho gradient-echo EPI *T*_2_*-weighted images depicting BOLD contrast for each session of the behavioral task (Poser et al., 2006). We used the following parameters for obtaining BOLD images: 30 axial slices (3.78 mm slice thickness), TR 2100 ms, TEs: 12/27.91/43.82/59.73 ms, flip angle 82°, FOV 14.4 × 14.4 cm, matrix 64 × 64, in-plane resolution 3.75 × 3.75 mm. Importantly, imaging at multiple echo times has the potential to increase sensitivity in brain regions that are typically subject to strong image distortions, including the inferior prefrontal cortex and temporal lobe (Poser et al., 2006). Each participant completed three sessions of the task, resulting in 1080 volumes per participant. After scanning, we combined images acquired with different TEs into a single image with optimal sensitivity by applying voxelwise weighted echo summation based on local *T*_2_* To improve localization of the functional data, a high-resolution anatomical scan was acquired during the same scan session (*T*_1_: MPRAGE; TR/TE 2.98/2300 ms, 1 × 1 voxels, slice thickness 1 mm, flip angle 9°, FOV 24 × 25.6 mm, 176 slices).

##### Behavioral analyses.

To determine whether dopamine modulated behavior on the task, we first investigated the effect of dopamine on task performance, the number of missed task trials, response time, and the distance between the initial appearance of the prediction bar and participants' final bid. Performance (error) was defined as the absolute difference between the mean of reward distributions and participants' predictions across all trials for each SD condition as the mean of the reward distribution would be the most accurate prediction on this task. All tests were conducted using parametric statistics (i.e., ANOVA's and Pearson correlations) because these variables were normally distributed.

##### Computational modeling of task behavior.

Detailed computational modeling of two independent datasets conducted previously showed that participants' behavior on this task can be successfully predicted using a variant of the Pearce-Hall (PH) reinforcement learning model (Pearce and Hall, 1980; Li et al., 2011) that scales PEs relative to reward variability (Diederen and Schultz, 2015; Diederen et al., 2016). This model performs particularly well as the PH dynamic learning rate enables participants to establish stable predictions in the face of continuing PEs, and the PE scaling relative to SD allows participants to restrain learning when PEs fluctuate more. We first sought to confirm whether the adaptive PH model (model 4, see below) also successfully predicted participants' behavior in the current study. With this aim, we used formal model comparisons (see below) to compare this model with a set of related reinforcement learning models (Diederen and Schultz, 2015; Diederen et al., 2016).

For each model, we consider the case in which participants' predictions (*y*) are assumed to result from a recursive generative process as follows:

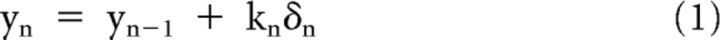
 Here, k_n_ denotes the learning rate and δ_n_ denotes the PE on trial *n*. The different reinforcement learning models varied in the calculation of the learning rate, which indicates the degree to which the PE on trial *n* is used to update the prediction on trial *n* + 1.

1. Rescorla-Wagner 1 (RW#1). We first consider the most basic reinforcement-learning rule: an RW model, in which participants update their predictions as a constant fraction, termed the learning rate, of the PE (Rescorla and Wagner, 1972):

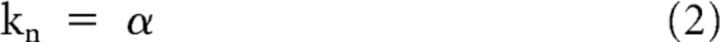
 2. RW#2. As a number of studies have reported a selective effect of dopaminergic agents on learning from positive outcomes (Pessiglione et al., 2006; van der Schaaf et al., 2014), we subsequently implemented an RW model (Rescorla and Wagner, 1972) with separate learning rates for positive and negative PEs to participants' prediction sequences, in keeping with previous work (Diederen et al., 2016) as follows:

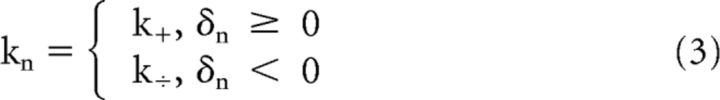
 where k_+_ and k_÷_ are the asymmetric RW learning rates.

3. PH#1. We subsequently compared this model with a PH model with a decreasing learning rate, which enables participants to achieve stable predictions in the phase of continuing PEs. A dynamic learning rate is essential when rewards are drawn from a Gaussian process as a constant (RW) learning rates interfere with the acquisition of stable predictions as follows:

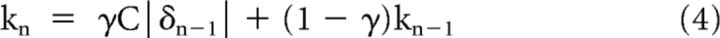
 Here, |δ| denotes the absolute PE, and C is an arbitrary scaling coefficient. The recursive process is initialized with the initial learning rate k_0_ = α. The learning rate depends on the absolute PE and learning rate on previous trials and on the decay constant γ.

4. PH#2. Finally, to account for the potential effect of SD in the PH model, we scaled the PE relative to log(SD) of the reward distributions, in line with previously documented procedures (Diederen and Schultz, 2015; Diederen et al., 2016) as follows:

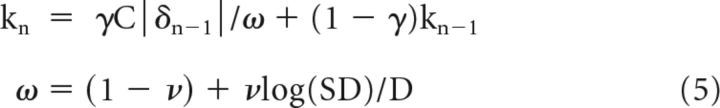
 Here, k_n_ denotes the learning rate on trial *n*, and C and D are arbitrary scaling coefficients. As previously, we estimated the extent of PE scaling (0 ≤ ν ≤ 1) for each participant across all SDs (Diederen and Schultz, 2015; Diederen et al., 2016). *v* = 0 indicates an absence of PE scaling, whereas *v* > 0 indicates the presence of PE scaling. k_1_ and γ are free parameters that are fitted to participants' prediction sequences. Importantly, previous work showed that this model outperformed other models as the PH dynamic learning rate enables participants to establish stable predictions in the face of continuing PEs, and the PE scaling relative to SD allows participants to restrain learning when PEs fluctuate more.

We fitted the free parameters Φ to the subjective predictions Y by maximizing the likelihood p(Y|Φ) = ∏_m_^M^ p(y_m_|Φ), where p(y_m_|Φ) = 𝒩(μ_m_, σ̂^2^), and Y = [y_1_ y_2_ . . y_M_] are the subjective predictions. We used a combination of nonlinear optimization algorithms implemented in MATLAB to estimate the free parameters to each participant's full dataset over the trials of all conditions. The parameters from the winning model were subsequently extracted and analyzed for drug effects.

To determine which learning parameters, derived from the best performing reinforcement learning model, might have affected learning performance, we performed a linear regression analysis with overall performance error (i.e., averaged across all conditions) as the dependent variable, estimated learning parameters as independent variables, and treatment group as a covariate.

##### fMRI preprocessing.

Statistical parametric mapping (SPM8; Wellcome Department of Cognitive Neurology, London) and MATLAB were used to analyze fMRI data. Preprocessing included within-subject image realignment, voxelwise weighted echo combination (summation based on local *T*_2_* measurements) (Poser et al., 2006), coregistration of functional images with the T_1_-weighted anatomical scan, spatial normalization to the MNI template in SPM8 (Ashburner and Friston, 2005), and spatial smoothing using an 8 mm FWHM Gaussian kernel for the ventral striatal region of interest (ROI; see below) and a 4 mm FWHM for the midbrain ROI (in keeping with the small size of this region). The time-series in each session were high-pass filtered (1/145 Hz), and serial autocorrelations were estimated using an AR(1) model.

##### fMRI first-level data analyses.

To examine adaptive coding, at the first level, a single regression model was created for each participant (Diederen et al., 2016). Cue onset, prediction onset, and reward onset were modeled as events of zero duration, separately for each SD condition. Reward onset events were modeled separately for trials with positive and negative PEs as BOLD responses in the human midbrain and striatum tend to be more pronounced for negative compared with positive PEs (D'Ardenne et al., 2008; Liu et al., 2011; Diederen et al., 2016). Reward onsets were parametrically modulated with trialwise reward outcome value and PE. The PE parametric modulator was orthogonalized with respect to the outcome value regressor to ensure that this parametric modulator captured BOLD responses that varied with PEs, independently of reward magnitude. Initial inspection of PE slopes confirmed an increase in the magnitude of PE slopes for positive compared with negative PEs in the midbrain (*T*_(56)_ = −2.19, *p* = 0.017) and ventral striatum (*T*_(56)_ = −1.77, *p* = 0.041) ROI (for a description of the ROIs, see below; Fig. 1*C*). Additional covariates were included for error trials (no response within 3500 ms) and the prediction time (response time from cue onset to prediction) in nonerror trials. All events of interest and covariates were convolved with the standard hemodynamic response in SPM8. Finally, the realignment parameters were included as regressors of no interest to model movement related artifacts. All regressors were fitted to the data using GLM estimation.

##### fMRI second-level data analyses.

A two-step approach was taken for the second-level analyses. In the first step, we determined whether the previously reported adaptive coding effect was replicated (Diederen et al., 2016) by examining the placebo group. In line with previous work (Diederen et al., 2016), adaptive PE coding was defined as an increase in PE regression slopes for smaller compared with larger SDs (SD5 > SD10 > SD15), reflecting a greater sensitivity to small changes in PEs in distributions with lower SDs (Kobayashi et al., 2010). As we have previously shown, this relationship (SD5 > SD10 > SD15) is nonlinear, and each level was therefore weighted by the inverse of the SD (Diederen et al., 2016). We then sought to examine the effect of dopaminergic manipulation on this adaptive coding effect. All analyses were restricted to the a priori ROIs of the midbrain and the ventral striatum. Additional exploratory whole-brain analyses were also performed.

##### ROIs: PE adaptation.

We have previously shown in an independent dataset that PEs are adaptively coded in the human midbrain (SN/VTA complex) and ventral striatum (Diederen et al., 2016). We therefore focused our main comparisons on these ROIs, and this is in line with previous studies investigating dopaminergic perturbations (Pessiglione et al., 2006; Chowdhury et al., 2013). ROI masks were created as spheres centered on the peak coordinates of clusters that previously showed robust PE adaptation in the midbrain (−8, −18, −10; 8, −18, −10 and ventral striatum (−18, 1, −10; 18, 1, −10) in an independent sample of healthy individuals who performed the same task (Diederen et al., 2016) (Fig. 1*D*). For the ROI spheres, the radii (6/8 mm for the midbrain/ventral striatum) were chosen to ensure that the spheres fell within the anatomical boundaries of the midbrain SN/VTA complex and the ventral striatum. We focused our main comparisons on these functional, rather than anatomical, ROIs because anatomical areas might contain multiple functional loci. However, to determine the robustness of any observed significant effects, we repeated ROI analyses using anatomical masks for the midbrain SN/VTA complex and the ventral striatum. The SN/VTA complex was drawn on a normalized high resolution magnetic transfer image acquired using the same MRI scanner as the functional MR images (Gruber et al., 2014). For the anatomical definition of the ventral striatum, we used a mask of the nucleus accumbens as included in the IBASPM toolbox (Aleman-Gomez et al., 2006).

##### ROIs: instructional cues signaling reward variability.

As we had no strong a priori hypotheses about brain areas encoding the instructional cues that predicted reward variability, we explored the effect of dopaminergic modulation on the neural responses to the cues using a leave-one-subject-out approach (Esterman et al., 2010). We restricted the analysis to a set of anatomical regions that have been implicated in the signaling of instructional cues including cues on reward variability (Preuschoff et al., 2006; Atlas et al., 2016), namely, the insula, anterior cingulate cortex (ACC) and middle frontal gyrus (MFG). In the leave-one-out approach, a single subject is iteratively left out of the first-stage group analysis. The resulting group analyses return ROIs that serve as an independent functional localizer for the subject left out. The peak coordinates in the insula, ACC and MFG, for each (left-out) subject were used to define spherical ROIs of 8 mm diameter for that subject.

##### Examination of adaptive coding in the placebo group.

Linear contrasts on regression coefficients of interest from the first level were entered into a second-level, random effects, repeated-measures ANOVA. The key contrast of interest was the main effect of PE adaptation (SD5 > SD10 > SD15) as a nonlinear contrast weighted by SD^−1^ (Diederen et al., 2016). This contrast revealed regions where BOLD responses to positive and negative PEs varied more strongly with PEs when the SD was smaller, independent of outcome value. For these analyses, we applied small-volume corrections (SVCs) in SPM8 with the midbrain and ventral striatum combined into one ROI, even though we used different smoothing kernels for these regions, to ensure that corrections for multiple comparisons were conducted across all voxels in both areas. For the SVCs, we considered activations significant at *p* < 0.05 family-wise error (FWE) corrected. For completeness, we also explored whole brain effects of adaptive PE coding in the placebo group, and these results are reported at *p* < 0.05, FWE corrected at both the cluster and voxel level.

##### Examination of dopaminergic modulation of adaptive coding.

For the between-group ROI analyses, the adaptive coding contrast (SD5 > SD10 > SD15, nonlinear contrast weighted by SD^−1^) was generated at the first level for each participant across both positive and negative PEs. Parameter values for this contrast were extracted and averaged across all voxels in the ROIs using MATLAB scripts. The extracted parameter estimates were entered into subsequent statistical analyses in MATLAB. As these measures were not normally distributed, the between-group comparisons were conducted using nonparametric tests. To limit the number of multiple comparisons, we only used *post hoc* tests between the placebo group and each of the experimental groups. Thus, the Bonferroni-corrected threshold for significance was *p* < 0.025 for all *post hoc* tests.

To examine whether there was a selective modulation of positive prediction error coding in the ventral striatum by dopaminergic agents, we examined this using an adaptive contrast as above, but restricted to the positive PEs.

To investigate whether increases in adaptive PE coding in the midbrain or ventral striatum were associated with improvements in task performance, we calculated Spearman correlations between adaptive PE coding and overall performance error.

##### Working memory and dopaminergic modulation.

As previous studies have shown that baseline working memory performance can mediate the influence of dopaminergic medication on the neural correlates of cognitive tasks (Kimberg et al., 1997; van der Schaaf et al., 2014), we examined whether working memory capacity (estimated using the digit span backwards) mediated behavioral and neural adaptation to reward variability. For the behavioral adaptation, we conducted an additional analysis that included working memory as a covariate. For the neural data, we calculated simple nonparametric (i.e., Spearman) correlations between working memory and adaptive coding in the midbrain and ventral striatum as the neural data did not meet assumptions for normality.

## Results

Fifty-eight participants were included in the behavioral analyses and 57 in the fMRI analyses (19 per group). Complete data were unavailable for 5 participants due to >30% missed task trials (*N* = 1; bromocriptine group), nausea (*N* = 1; sulpiride group), back pain (*N* = 1, sulpiride group), anxiety (*N* = 1; sulpiride group), and neck pain and MRI reconstruction problems (*N* = 1; placebo group). These 5 participants were excluded from all analyses, and an additional participant was excluded from the fMRI analyses because of left-handedness (*N* = 1; placebo group). The included participants in each group were matched for age, sex, years of education, working memory capacity as assessed with the Wechsler reverse Digit Span task (Wechsler, 1958), verbal IQ assessed using the National Adult Reading Test (Nelson and Willison, 1991), and BMI (Table 1). In addition, in each group participants experienced similar changes in mood between dosing and MRI data acquisition (Bond and Lader, 1974) (Table 1). None of the participants experienced significant extrapyramidal side effects as assessed by a trained psychiatrist using the Simpson Angus scale (Simpson and Angus, 1970). However, due to fMRI acquisition issues, the time between dosing and the start of the fMRI scans differed on trend level (*p* = 0.099) between the three groups (Table 1). When only the two active drug groups were compared, this difference was significant (χ^2^_(36)_ = 4.08, *p* = 0.0434). To control for the timing of dosing, we quantified and removed the variance explained by this variable using simple regressions. Specifically, the time between dosing and the start of the fMRI scans was the predictor, and the outcome variable of interest was the dependent variable in these regressions. Subsequent group comparisons were conducted on the residuals of these regressions. We used this procedure for all behavioral and fMRI outcome variables, except for tests comparing percentages.

**Table 1.** Demographic information of the participant groups*^a^*

	Bromocriptine (*N* = 19)		Placebo (*N* = 19)		Sulpiride (*N* = 19)		*F*(χ^2^)	*p* value
	Mean	SEM	Mean	SEM	Mean	SEM		
Age	24.00	0.91	23.9	1.1	24.9	1.0	0.27	0.77
Time dosing fMRI	168.5	2.1	166.9	2.1	160.8	3.5	2.35	0.10
Gender	10 M	9 F	9 M	10 F	11 M	8 F	0.42	0.81
BMI	24.5	1.0	22.4	1.0	22.7	0.6	1.65	0.20
Education (years)	14.8	0.5	15.1	0.5	14.7	0.6	0.17	0.84
NART	18.9	1.8	16.1	1.4	18.7	2.1	0.73	0.48
Digit span	6.8	0.9	6.1	0.3	5.8	0.3	0.71	0.50
BL alertness	−1.7	1.0	−1.2	0.7	−1.7	1.3	0.10	0.90
BL calmness	0.1	0.4	−0.4	0.9	0.5	0.5	0.43	0.65
BL contentedness	0.7	0.3	−0.4	0.5	0.3	0.6	1.52	0.23

*^a^*NART, National Adult Reading Test; BL, Bond and Lager. SEM, Standard error of the mean.

### Task performance

**Figure 2.**
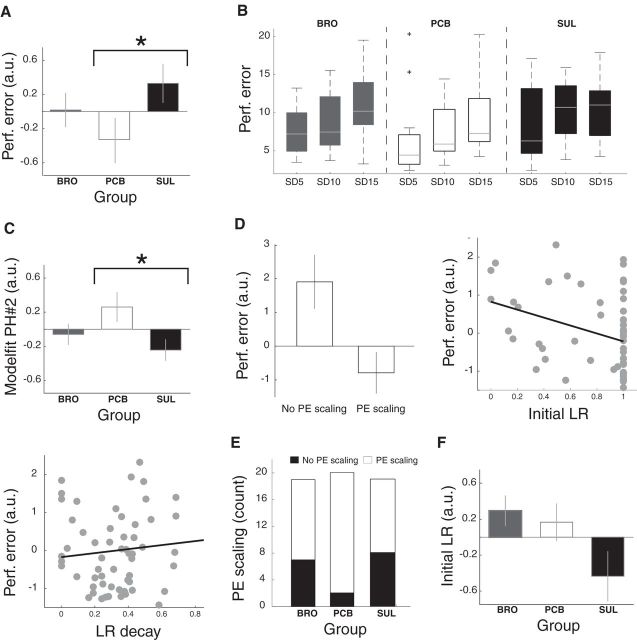
***A***, Average (±SEM) performance error for each of the experimental groups and SD conditions. Performance error was significantly increased in the sulpiride group compared with the placebo group, indicating that sulpiride impairs performance. ***B***, There was no significant interaction between SD and treatment group on average (±SEM) performance error. ***C***, Average (±SEM) predictions generated under the adaptive PH model (PH#2) more closely tracked participants' prediction sequences in the placebo compared with the sulpiride group. ***D***, Fitted PE scaling (top left), initial learning rate (top right), and the decay in learning rate (bottom left) were all predictive of overall performance error as identified using regression analysis. ***E***, The proportion of participants that scaled their PEs relative to reward variability (scaling parameter > 0) was significantly decreased in the sulpiride group compared with placebo. ***F***, The average (±SEM) initial learning rate differed on trend level between the groups. Lower performance error indicates higher performance. *y* axes indicate standardized (i.e., *z*-scored) residual outcome variables, after correction for the time between dosing and the start of the fMRI scan, as this time differed between the treatment groups (except for ***B***, ***D***, top left). ***B***, Raw performance data. ***D***, Left, Based on a simple subject count). * indicates significance. a.u., arbitrary units; BRO, bromocriptine; LR, learning rate; PCB, placebo; PE, prediction error; Perf., performance; PH, Pearce-Hall; SD, standard deviation; SUL, sulpiride.

To determine whether the dopaminergic manipulations affected task performance, we first inspected participants' performance error, quantified as the absolute difference between each participant's predictions and the mean of the reward distributions across all three SD conditions. Importantly, performance error was significantly modulated by dopaminergic perturbation (*F*_(2,165)_ = 5.41, *p* = 0.005; Fig. 2*A*), and *post hoc* testing revealed that performance was significantly reduced in the sulpiride group compared with placebo (*p* = 0.003), whereas bromocriptine decreased performance on trend level (*p* = 0.072). When the SD conditions were considered separately, performance error monotonically increased with SD in the placebo and bromocriptine groups, but this distinction was less clear in the sulpiride group (Fig. 2*B*). However, this effect was not statistically significant (i.e., the SD × treatment group interaction was not significant; *F*_(4,165)_ = 0.28, *p* = 0.89). However, it is important to note that performance error reflects the influence of multiple learning parameters and not PE scaling alone (see below).

The total number of missed task trials did not differ significantly between the experimental groups (*F*_(2,55)_ = 0.56 *p* = 0.576). It is therefore unlikely that differences in the number of missed trials account for the difference in task performance between the groups. To determine whether the dopaminergic effect on task performance could be related to subtle drug-induced motor symptoms, we inspected response times. As expected, response times varied with the distance between the initial point of the prediction bar on the scale (which was randomized) and participants' predictions (i.e., the scroll distance; *r* = 0.43, *p* < 0.001). After accounting for the effect of scroll distance, response times did not significantly vary with treatment group (*F*_(2,54)_ = 2.03, *p* = 0.141). In addition, scroll distance was similar for the three groups, thus suggesting that the dopaminergic agents did not influence participants' motivation to reveal their true prediction of reward (*F*_(2,55)_ = 2.37, *p* = 0.103).

### Computational modeling

Formal model comparisons using Akaike and Bayesian information criteria confirmed that participants' behavior was best fit by the adaptive PH model that includes a decay in learning rate across trials and scaling of PEs relative to the variability in reward (for model comparisons, see Table 2). Based on the superior fit of this model, we used the above parameters in subsequent behavioral analyses.

**Table 2.** Quality of the generative models fitted to behavioral data given as the mean difference (*d*) in criterion values (AIC and BIC) across participants*^a^*

Model	Criterion	RW#1	RW#2	PH#1
RW#2	*d*AIC	−6.5		
	*d*BIC	−3.3		
PH#1	*d*AIC	−11.7	−5.1	
	*d*BIC	−8.4	−5.1	
PH#2	*d*AIC	−15.4	−8.9	−3.8
	*d*BIC	−9.0	−5.7	−0.6

*^a^*RW#1, Rescorla-Wagner model with one learning rate fitted across positive and negative PEs; RW#2, Rescorla-Wagner model with separate learning rates for positive and negative PEs; PH#1, Pearce-Hall model with a fitted initial learning rate and a parameter guiding the trialwise decay in learning rate; PH#2, Adaptive Pearce-Hall model with a fitted initial learning rate and a parameter guiding the trialwise decay in learning rate; AIC, Akaike information criteria; BIC, Bayesian information criteria. Here, PEs are scaled relative to reward variability. Models are fitted across all trials, conditions, and participants.

Simple regressions were then performed to determine how closely predictions generated under the adaptive PH model tracked participants' prediction sequences in each group. A direct comparison of the groups revealed that under dopaminergic perturbation, the adaptive PH model (PH#2) did not predict participants' behavior as well as under placebo *F*_(2,54)_ = 3.28, *p* = 0.045. This effect was driven by a lower *R*^2^ (averaged over all task conditions) in the sulpiride group compared with placebo (*post hoc* tests: placebo vs sulpiride, *p* = 0.022; placebo vs bromocriptine, *p* = 0.136; Fig. 2*C*). Working memory capacity did not modulate the effect of dopaminergic perturbation on behavioral adaptation (*F*_(1,53)_ = 0.24, *p* = 0.624).

Under this model, the differences in the sulpiride group could relate to the SD scaling parameter, learning rate or decay (of learning rate) parameter, or a combination of these. The linear regression showed that the presence/absence of PE scaling (*p* = 0.014), initial learning rate (*p* = 0.004), and decay in learning rate (*p* = 0.042) all significantly impacted on performance error. Performance error decreased with the presence of PE scaling, higher initial learning rates, and lower decay in learning rate (Fig. 2*D*). A larger proportion of participants in the sulpiride group (8 of 19) did not scale PEs relative to SD, as indicated by the estimated scaling parameter (υ) of 0, compared with the placebo group (2/20 (χ^2^_(1)_ = 5.27, *p* = 0.0217; Fig. 2*E*). The learning rate differed on trend level between the sulpiride and the placebo group (*T*_(37)_ = 1.78, *p* = 0.084; Fig. 2*F*), and there were no differences in the decay parameter (*T*_(37)_ = 0.36, *p* = 0.718). This suggests that decreases in performance, as observed in the sulpiride group, are at least partially related to a failure to scale PEs to the variability in rewards.

Finally, we examined whether there was a selective effect of dopaminergic perturbation on learning from positive PEs. To this end, we first used the RW reinforcement-learning model with separate learning rates for positive and negative PEs to participants' prediction sequences (Pessiglione et al., 2006; Diederen et al., 2016). There was no significant interaction between group and the sign of the PE (*F*_(2,110)_ = 0.10, *p* = 0.905), and the learning rates for positive PEs did not differ between the treatment groups (*F*_(2,55)_ = 0.16, *p* = 0.855).

We then examined the decrease in learning rate across the SD conditions, which provides an alternative measure of behavioral adaptation (Diederen et al., 2016), separately for positive and negative PEs. Behavioral adaptation did not vary with the sign of the PE (i.e., the decrease in learning rate × PE sign *F*_(2,110)_ = 0.67, *p* = 0.512). Thus, the behavioral effect of dopamine on participants' behavior was not selective for positive PEs.

### Neural adaptation to reward variability

**Figure 3.**
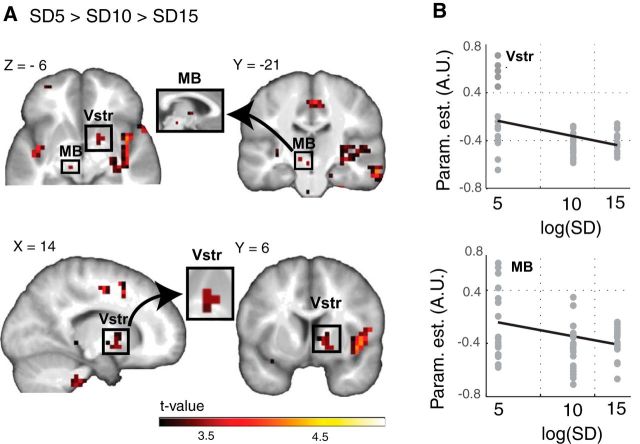
***A***, Adaptive PE coding in the placebo group (SD5 > SD10 > SD15; nonlinear contrast weighted by SD^−1^). For display purposes only, the maps shown in the figure are based on a whole-brain threshold of *p* < 0.001 uncorrected for multiple comparisons. ***B***, Average (±SEM) PE regression slopes in the placebo group for the midbrain and ventral striatum. A.U., arbitrary units; MB, Midbrain; Param.est., Parameter estimates; PE, prediction error; SD, standard deviation; SEM, standard error of the mean; Vstr, Ventral striatum.

We first sought to replicate our previous findings on adaptive PE coding in the placebo group. Ventral striatal and midbrain activity increased with increases in PE magnitude in the SD5 conditions compared with SD10 and SD15 conditions, in line with the notion of adaptive coding (16, 0, −6, *Z* = 3.17, *p* < 0.05 FWE, SVC and −3, −22, −10, *Z* = 3.13, *p* < 0.05 FWE SVC for the ventral striatum and midbrain, respectively; Fig. 3*A*,*B*). Whole-brain analyses (*p* < 0.05 cluster level) revealed additional adaptive coding in three clusters, including the superior temporal gyrus, the claustrum and insula, the lentiform nucleus, the thalamus, the cingulate gyrus, and the MFG (Fig. 3*A*; Table 3). These findings are highly comparable with those previously reported in an independent dataset (Diederen et al., 2016), suggesting that the adaptive effect is replicable and robust. When we repeated these analyses across all of the groups, no significant effect of adaptation could be observed in either the a priori defined ROIs or on whole brain (all *p* values > 0.1).

**Table 3.** Whole-brain adaptive coding in the placebo group*^a^*

Brain area	Cluster size	Maximum *T* value	Cluster *p* value	MNI coordinates		
				*x*	*y*	*z*
Right inferior temporal gyrus	104	4.37	0.01	65	−15	−26
Right middle temporal gyrus				65	−22	−18
Right claustrum				42	4	−6
Right insula				46	−15	−10
Right lentiform nucleus				31	−18	−2
Left subgyral	117	4.09	0.05	−44	−11	−14
Left superior temporal gyrus				−37	−3	−18
Left thalamus				−26	−30	2
Left claustrum				−33	−26	2
Left superior temporal gyrus				−44	−41	6
Right paracentral lobule	62	4.13	0.05	5	−18	50
Left cingulate gyrus				−3	−7	46
Left paracentral lobule				−3	−7	46
Right medial frontal gyrus				16	−7	50

*^a^*Cluster sizes, *p* values, *t* values, and locations of local maxima for brain regions, other than the midbrain (SN/VTA complex) and ventral striatum, showing adaptive coding of PEs to reward variability. MNI, Montreal Neurological Institute; PE, prediction error; SN, Substantia nigra; VTA, Ventral tegmental area.

#### 

##### Dopaminergic perturbation modulates adaptive coding.

**Figure 4.**
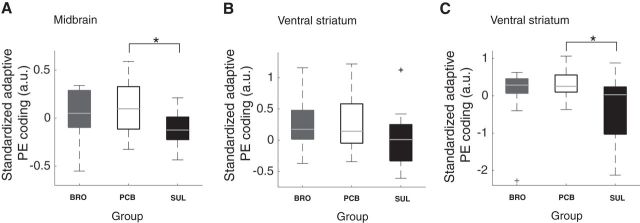
***A***, Median and range of adaptive PE coding in midbrain ROI. Sulpiride significantly perturbed adaptive coding in the midbrain ROI. ***B***, Median and range of adaptive PE coding in the ventral striatal ROI. ***C***, Medium and range of adaptive coding of positive PEs in the ventral striatal ROI. Whereas dopamine did not perturb PE coding across positive and negative PEs in the ventral striatum, there was a selective effect of dopamine on positive PEs in this ROI. Each boxplot represents standardized (i.e., *z*-scored) residual adaptive coding values after correction for the time between dosing and the start of the fMRI scan as this time differed between the treatment groups (for details, see Results). Thus, higher values on the *y*-axis indicate an increase in adaptive coding after adjusting the data for the effect of the time between dosing and the start of the fMRI scan. * indicates significance. a.u., arbitrary units; BRO, bromocriptine; PCB, placebo; PE, prediction error; SUL, sulpiride.

Here we examined the adaptive coding contrast (SD5 > SD10 > SD15; nonlinear contrast weighted by SD^−1^) from all participants. Increases in this contrast indicate increases in the differential effect of SD on PE coding and suggest a greater sensitivity to changes in SD. An ROI analysis on the adaptive coding parameter estimates confirmed the presence of significant adaptive coding in the placebo group in both the midbrain (*z* = 1.73, *p* = 0.04) and the ventral striatum (*z* = 2.33, *p* = 0.01; Fig. 4). Direct comparisons of the adaptive coding contrasts across the three groups showed that dopaminergic perturbation significantly altered adaptive PE coding in the midbrain (χ^2^_(2,54)_ = 8.26, *p* = 0.016; Fig. 4*A*), but only on trend level in the ventral striatum (χ^2^_(2,54)_ = 4.62, *p* = 0.099; Fig. 4*B*). *Post hoc* tests revealed that the effect in the midbrain was driven by sulpiride and that bromocriptine did not alter adaptive coding (*p* = 0.005/*p* = 0.55 for sulpiride/bromocriptine vs placebo). Additional analysis using an anatomical definition of the SN/VTA complex (see Materials and Methods) confirmed reduced midbrain adaptive PE coding in the sulpiride compared with the placebo group (χ^2^_(1,36)_= 4.6, *p* = 0.032).

As the effect of dopaminergic modulation in the ventral striatum has been shown to be more selective for positive PEs, we examined the adaptive coding of positive PEs alone. These analyses revealed significant alteration in ventral striatal adaptation for positive PEs (χ^2^_(2,54)_ = 6.07, *p* = 0.048; Fig. 4*C*), whereas adaptation for negative PEs was unaltered by dopamine (χ^2^_(2,54)_ = 0.65, *p* = 0.724). This effect on positive PEs was driven by a decrease in adaptive coding of positive PEs in the sulpiride group (*p* = 0.026), whereas bromocriptine did not affect adaptive coding of PEs in the ventral striatum (*p* = 0.75). Parameters extracted from an anatomical definition of the substantia nigra confirmed this result (χ^2^_(1,36)_= 4.24, *p* = 0.040). Thus, these results suggest that sulpiride perturbed adaptive prediction coding across positive and negative PEs in the midbrain and for positive PEs alone in the ventral striatum.

Midbrain adaptive coding did not vary with working memory performance in either the bromocriptine (ρ = −0.09, *p* = 0.71) or the sulpiride group (ρ = −0.11, *p* = 0.67). Similarly, we observed no significant correlations between working memory and adaptive coding of positive PEs in the ventral striatum for the bromocriptine group (ρ = −0.08, *p* = 0.74) or the sulpiride group (ρ = −0.07, *p* = 0.78), suggesting that baseline working memory capacity does not mediate adaptive coding. In addition, we observed no significant relationship between performance error and adaptive PE coding in the midbrain (ρ = −0.1348, *p* = 0.150) and ventral striatum (ρ = −0.1117, *p* = 0.199).

For completeness, we subsequently explored the effect of dopaminergic medication on whole-brain adaptive coding; no such effects could be observed (all *p* values > 0.1). In addition, dopamine did not significantly impact on overall PE coding (averaged across the SD conditions) on whole-brain level (all *p* values > 0.1). Similarly, ROI analyses revealed no significant effect of dopaminergic medication on overall PE coding in the midbrain (χ^2^_(2,54)_ = 4.43, *p* = 0.11) or ventral striatum (χ^2^_(2,54)_ = 0.65, *p* = 0.724) across positive and negative PEs. However, there was a trend-level effect of dopaminergic treatment on overall positive PE coding in the ventral striatum (χ^2^_(2,54)_ = 5.05, *p* = 0.08). This effect was driven by decreased overall positive PE coding in the supiride group compared with placebo (*p* = 0.03), whereas bromocriptine did not affect nonscaled positive PE coding in the ventral striatum (*p* = 0.75). These results suggest that dopaminergic perturbation selectively affected adaptation of PEs in this task.

### BOLD responses to cues signaling reward variability

**Figure 5.**
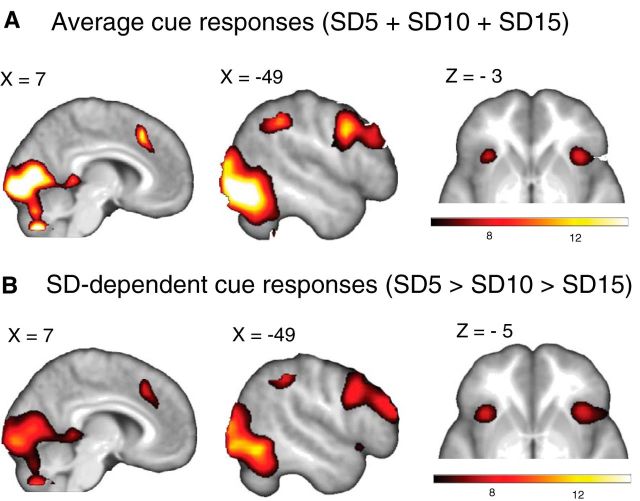
***A***, Bold response to cues signaling reward variability across the three groups. ***B***, Increased BOLD responses to cues signaling smaller reward variability (SD5 > SD10 > SD15; nonlinear contrast weighted by SD^−1^). We used a stringent initial threshold of *p* < 1e −11 combined with a minimal cluster size of 5 adjacent voxels as the cue event was associated with very strong signal changes. The cluster threshold was *p* < 0.05 FWE corrected for multiple comparisons. SD, standard deviation.

Across the three groups, cue onset averaged across SD conditions was associated with widespread activation in a network of regions, including the bilateral insula, the ACC/medial frontal gyrus, the MFG, and the cerebellum extending into the occipital lobe (for an overview of all significant loci, see Table 4; Fig. 5*A*). Using a nonlinear contrast analogous to the adaptive PE coding contrast (i.e., (SD5 > SD10 > SD15; weighted by SD^−1^), we found that in most of these regions the BOLD responses to the instructional cues increased as reward variability decreased (for all significant loci, see Table 4; Fig. 5*B*), These results suggest that participants attentively processed the cues before predicting the expected magnitude of upcoming rewards.

**Table 4.** BOLD responses to cues signaling reward variability and reward variability as a function of SD*^a^*

Brain area	Cluster size	Maximum *T* value	Cluster *p* value	MNI coordinates		
				*x*	*y*	*z*
BOLD responses to cues signaling reward variability						
Left declive (cerebellum)	3816	18.9	<0.001	−37	−74	−22
Right declive (cerebellum)		16.41	<0.001	38	−67	−22
Left precentral gyrus	137	12.1	<0.001	−44	4	34
Left MFG		9.79	<0.001	−52	30	34
Right MFG	421	11.47	<0.001	34	12	50
Right MFG		10.85	<0.001	50	42	22
Right precentral gyrus		10.43	<0.001	50	12	34
ACC/medial frontal gyrus	36	9.69	<0.001	4	23	46
Left thalamus	13	8.6	<0.001	−22	−33	−2
Right insula	26	8.5	<0.001	38	19	−6
Left MFG	6	8.04	<0.001	−29	49	6
Left insula/claustrum	5	7.94	<0.001	−33	19	−2
BOLD responses to cues signaling reward variability as a function of SD						
Left declive (cerebellum)	3081	17.95	<0.001	−37	−74	−22
Right declive (cerebellum)		15.21	<0.001	38	−67	−22
Left precentral gyrus	71	11.18	<0.001	−44	4	34
Right MFG	278	10.41	<0.001	50	46	18
Right precentral gyrus		9.89	<0.001	50	12	34
Right inferior semilunar lobule (cerebellum)	50	10.03	<0.001	8	−74	−42
Left inferior semilunar lobule (cerebellum)		9.99	<0.001	−14	−74	−46
Left thalamus	16	9.08	<0.001	−22	−33	−2
Left supramarginal gyrus	16	8.92	<0.001	−48	−44	38
Right thalamus	27	8.92	<0.001	20	−33	−2
ACC/medial frontal gyrus	16	8.75	<0.001	4	23	42
Left insula/claustrum	11	8.63	<0.001	−33	19	−2
Right insula	23	8.11	<0.001	38	19	−6
Left MFG	5	8.09	<0.001	−52	30	34

*^a^*Cluster sizes, *p* values, *z* values, and locations of local maxima for brain regions showing increases in BOLD responses to the instructional cues as a function of decreases in SD We used a stringent initial threshold of *p* < 1e −11 combined with a minimal cluster size of 5 adjacent voxels as the cue event was associated with very strong signal changes. The cluster threshold was *p* < 0.05 FWE corrected for multiple comparisons. MNI, Montreal Neurological Institute.

### Effect of dopaminergic modulation on BOLD responses to cues

**Figure 6.**
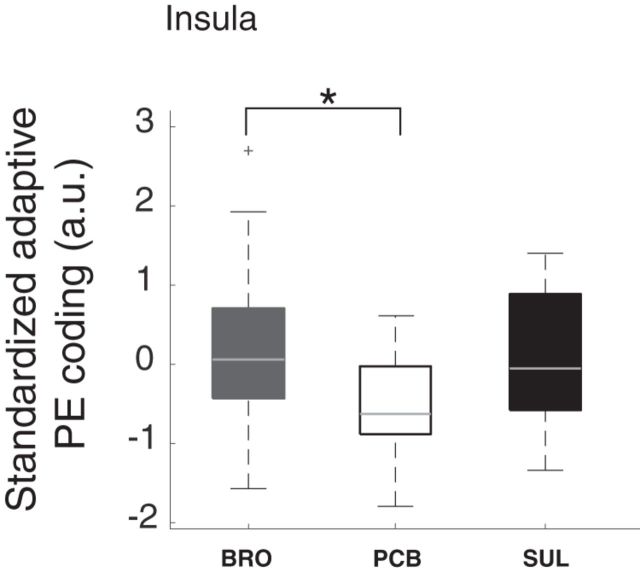
Median and range of average BOLD responses to the cues predicting reward variability. The dopaminergic modulation perturbed responses to the reward variability predicting cues on trend level. This effect was driven by a difference between the bromocriptine and the placebo group. Boxplot represents standardized (i.e., *z*-scored) residual adaptive coding values after correction for the time between dosing and the start of the fMRI scan as this time differed between the treatment groups (for details, see Results). Thus, higher values on the *y*-axis indicate an increase in BOLD responses to the reward predicting cues after adjusting the data for the effect of the time between dosing and the start of the fMRI scan. * indicates significance. a.u., arbitrary units; BRO, bromocriptine; PCB, placebo; PE, prediction error; SUL, sulpiride.

ROI analyses using a leave-one-out approach (see Materials and Methods) revealed a trend-level effect (required *p* value after Bonferroni correction for the 3 ROIs = 0.0167) in the insula (χ^2^_(2,54)_ = 7.9, *p* = 0.019; Fig. 6), but not in the ACC (χ^2^_(2,54)_ = 1.14, *p* = 0.57) and the MFG (χ^2^_(2,54)_ = 0.41, *p* = 0.57). *Post hoc* tests indicated that the trend-level effect in the insula was driven by increased responses in the bromocriptine and sulpiride groups compared with the placebo group (*p* = 0.017/*p* = 0.031 for bromocriptine/ sulpiride vs placebo). However, the difference between the sulpiride and placebo groups did not survive the multiple comparisons Bonferroni correction threshold of 0.025. No significant effect of dopamine was seen on the relationship between the response to the cue and SD in the insula (χ^2^_(2,54)_ = 4.27, *p* = 0.12), the ACC (χ^2^_(2,54)_ = 0.42, *p* = 0.81), and the MFG (χ^2^_(2,54)_ = 0.94, *p* = 0.62), suggesting that all groups were equally sensitive to cued differences in reward variability.

## Discussion

We sought to examine the effect of dopaminergic perturbation on PE adaptation to reward variability. We used a validated paradigm that requires participants to code PEs relative to SD, in conjunction with pharmacological perturbations of dopaminergic transmission. The dopamine antagonist sulpiride reduced adaptive PE coding in the midbrain, and for positive PEs alone in the ventral striatum. Sulpiride also perturbed overall performance, and computational modeling suggested that this was partially driven by a decrease in PE scaling, although we did not observe a differential effect of SD on raw performance data. These findings suggest that normal dopaminergic function is critical for adaptive PE coding, in line with previous work that demonstrated that monkey midbrain dopamine neurons code PEs relative to the distribution of predicted reward (Tobler et al., 2005). Although previous observations of adaptive coding in the human midbrain and striatum strongly suggested a role for dopamine in the adaptive process (Bunzeck et al., 2010; Park et al., 2012; Diederen et al., 2016), to our knowledge this is the first demonstration of this role of dopamine in humans.

These findings extend our understanding of the role of dopamine in PE signaling and error-driven learning to include its adaptive coding function. The former roles have been well demonstrated in studies of individuals treated with the dopamine precursor l-DOPA, which showed that enhancing dopamine transmission can increase learning rates, task performance and striatal PE activity (Pessiglione et al., 2006; Chowdhury et al., 2013; Rutledge et al., 2009). We observed no significant effect of the dopaminergic perturbation on unscaled PE coding, which might seem at odds with previous work (Pessiglione et al., 2006; Chowdhury et al., 2013). It is, however, conceivable that the seeming discrepancy in findings relates to the nature of the used tasks. Previous studies used experimental paradigms in which the unscaled PEs served as the learning signal, whereas the scaled PE is the (crucial) learning signal in our task. As dopamine is involved in efficient PE coding, we contend that, in this paradigm, dopaminergic manipulation would affect adaptively coded, rather than unscaled, PEs (Tobler et al., 2005).

In real-world situations where outcomes can be variable, it is critical to code PEs relative to variability. Such adaptive coding would be beneficial for learning as supported by the observation that increases in adaptive coding correlate with performance improvements (Diederen et al., 2016). Adaptive coding is a ubiquitous property of the brain and has been observed across perceptual systems (Carandini and Heeger, 2011) and to reward responses (Nieuwenhuis et al., 2005; Elliott et al., 2008; Padoa-Schioppa, 2009; Kobayashi et al., 2010; Cox and Kable, 2014). Adaptive coding makes optimal use of neurons' limited dynamic firing range and thus facilitates optimal sensitivity to fluctuations in outcomes (Kobayashi et al., 2010).

The effect of dopaminergic perturbation on PE coding in the human midbrain has not been previously reported, presumably because most studies restricted their comparisons to the striatum. Although D2 receptor density is highest in the basal ganglia, the midbrain contains D2 (auto)receptors, which exert inhibitory control on midbrain dopamine neurons (Aghajanian and Bunney, 1977; Lacey et al., 1987; Mercuri et al., 1992). It is unclear, however, how antagonism of midbrain autoreceptors may result in attenuation of adaptive PE coding. A speculative possibility is that partial autoreceptor blockade produces an initial increase in dopamine firing that leads to greater activation of the inhibitory autoceptors via collaterals that feedback into the soma or other nearby cells, producing a net decrease in dopaminergic firing (Deutch et al., 1988; Bayer and Pickel, 1990). However, blockade of autoreceptors could also lead to increases in dopamine (Frank and O'Reilly, 2006). Whereas the latter might be expected to result in improved adaptive coding, increased dopamine could also lead to impaired adaptive coding as an optimum level of dopamine is required for successful cognitive functioning (Cools and D'Esposito, 2011).

In the ventral striatum, the dopaminergic effect was selective for positive PEs, in keeping with the finding that l-DOPA affected striatal PE coding for reward but not losses (Pessiglione et al., 2006). Furthermore, some studies showed that patients with Parkinson's disease learn better to avoid negative outcomes than to obtain positive outcomes (Frank et al., 2004; Cools et al., 2006), which is remediated by dopamine enhancing medication that selectively improves learning from positive outcomes (Frank et al., 2004; Bódi et al., 2009; Rutledge et al., 2009). Conversely, sulpiride can affect reversal learning and choice performance for positive outcomes in healthy participants (Eisenegger et al., 2014; van der Schaaf et al., 2014). In contrast to these studies, we did not observe a behavioral effect of learning from positive versus negative PEs. Differences between behavioral and neural adaptation may reflect increased sensitivity of fMRI analyses (Wilkinson and Halligan, 2004). Alternatively, the effects of dopamine on behavior may be more closely related to midbrain instead of striatal responses. Differences in PE coding between the midbrain and ventral striatum have been reported previously (O'Doherty et al., 2006; D'Ardenne et al., 2008; Klein-Flügge et al., 2011) and are typically interpreted to result from the fact that striatal PE representations are not exclusively mediated by an afferent dopaminergic signal (Daw et al., 2006; Haber, 2011). It is less clear, however, why these differences became apparent under dopaminergic modulation. It should also be noted that the selective effect of sulpiride on the adaptation of positive PEs in the ventral striatum was identified using direct, a priori planned, comparisons, rather than from a significant interaction. This result should therefore be interpreted with caution.

We did not see an effect of dopaminergic perturbation on the instructional cues, which might suggest that dopamine did not affect the estimation of reward variability, but rather impaired scaling of PEs relative to variability. Targeted studies are needed to account more precisely for the lack in PE scaling. In addition, we observed no significant correlations between performance and adaptive PE coding in contrast to previous work (Diederen et al., 2016). This difference in findings may relate to additional noise induced by the pharmacological manipulation in the behavioral and neural measures, which may have obscured the presence of a significant correlation.

There are limitations of the pharmacological dopaminergic approach. There is debate regarding the directionality of perturbation as some studies showed improved, rather than impaired, task performance following administration of D2 antagonists (Jocham et al., 2011; van der Schaaf et al., 2014). Such seemingly incongruent results are thought to result from interindividuality in baseline dopamine levels and a preponderance of presynaptic over postsynaptic D2 blockade (Cools and D'Esposito, 2011). The effects vary with drug dose, drug serum levels, baseline dopamine capacity, and the genetically determined density of D2 receptors (Cools et al., 2009; Eisenegger et al., 2014).

Whereas sulpiride significantly altered adaptive PE coding, and task performance, bromocriptine did not impact these measures. It is possible that large interindividual variability in baseline dopamine levels obscured the effect of bromocriptine (Cools et al., 2009). Bromocriptine can improve learning in individuals with low baseline dopamine synthesis capacity while impairing it in subjects with high baseline dopamine synthesis capacity (Cools et al., 2009). Although we observed considerable variability in the bromocriptine group, our sample was of insufficient size to distinguish responders from nonresponders. One approach to deal with interindividual variability is stratification of drug effects by working memory (Kimberg et al., 1997; van der Schaaf et al., 2014). However, we did not find such a relationship. Another possibility for the absence of a bromocriptine effect is the high dose used. Studies that observed a significant effect of bromocriptine typically used lower doses (Mehta et al., 2001; Morcom et al., 2010; Medic et al., 2014). Indeed, Luciana and Collins (1997) observed significant improvements in performance on spatial working memory when participants were administered 1.25 mg of bromocriptine, but not when they received 2.5 mg. Finally, the absence of a significant effect of bromocriptine has been observed across different tasks, including reversal learning (van der Schaaf et al., 2014), perceptual decision-making (Winkel et al., 2012), and working memory (Luciana and Collins, 1997).

The observed role of dopamine in adaptive PE coding furthermore suggests that a breakdown of adaptation could result in inefficient learning in conditions associated with dopaminergic disturbance, such as psychosis (Fletcher and Frith, 2009). Although psychotic patients show aberrant PE signaling (Murray et al., 2008), future studies are required to determine whether adaptive PE coding is aberrant in individuals with delusional beliefs.

Another important avenue for future research would be to compare the role of dopamine in variable versus volatile environments. Whereas individuals' expectations should be robust in variable environments once learning has been completed, participants should flexibly update their predictions when outcomes originate from a volatile environment (Nassar et al., 2010).

Finally, it should be noted that recent work suggests that dopamine might encode the precision of information used to guide actions (Galea et al., 2012; Zokaei et al., 2012; Friston et al., 2014). This differs somewhat from our findings as we observed a role for dopamine in precision-weighted PE coding, not the encoding of precision itself.
